# Ill and substance-abusing parents: how can the general practitioner help their children? A qualitative study

**DOI:** 10.1186/s12875-016-0553-5

**Published:** 2016-11-08

**Authors:** Frøydis Gullbrå, Tone Smith-Sivertsen, Guri Rortveit, Norman Anderssen, Marit Hafting

**Affiliations:** 1 Research Unit for General Practice, Uni Research Health, Bergen, Norway; 2 Department of Global Public Health and Primary Care, University of Bergen, Bergen, Norway; 3 Department of Psychosocial Science, University of Bergen, Bergen, Norway; 4 Regional Centre for Child and Youth Mental Health and Child Welfare, Uni Research Health, Bergen, Norway

**Keywords:** General Practice, Family health, Parenting, Child of impaired parent, Qualitative research

## Abstract

**Background:**

Severe illness among parents may interfere with their parenting. Children having ill or substance-abusing parents are at risk of own health problems and psychosocial difficulties. The health care system should identify families in need of help and provide the help needed. For ill parents, it can be difficult to seek help and advices for their parenting. The aim of this study was to identify important factors for the general practitioner (GP) to bear in mind during encounters with ill and substance-abusing parents, to enable the GP to provide appropriate support for the children.

**Method:**

A qualitative approach was chosen and the data material was semi-structured individual interviews with 12 parents with mental illness, substance abuse or severe somatic illness. The participants were recruited through GPs in Norway, and the interviews were performed in 2014. We used systematic text condensation for analysis.

**Results:**

It was important for the participants that the GP was oriented about their family and children’s situation. They wanted to be regarded as competent parents in ordinary families; however, they were aware that their illness affected their parenting. They expressed a need for advice about how to inform the children of their illness and talk to them about their challenges, and, if necessary, utilize helpers who could inform the children and talk to them directly. There were often many agencies involved, and it was important that the helpers cooperated and shared information. In addition, the parents were in need of information about support services.

**Conclusion:**

Ill parents in this study conveyed a double message to their helpers. They wanted to be considered as responsible and well-intended parents who wished the best for their children. At the same time they needed support in parenting. The GP should take the time to listen to the parents’ first spontaneous description about an ordinary daily life (while realising that it may not necessarily be an accurate report), then explore their worries and needs of support.

## Background

When parents suffer from severe somatic disease, mental illness or substance abuse, it may influence the caregiving of their children [1–4]. The impact of their problems on the children’s well-being depends on the specific situation of the family, i.e. whether there are other caregivers available to meet the children’s needs, or whether there is access to a support network [5]. Reports on the help-seeking behaviour among seriously ill parents regarding parental problems indicate that parents with substance abuse problems and mental illness are reluctant to seek such help. For some, this is because they are afraid of losing their parental rights [6, 7]. Studies from the UK and Norway have found that parents with serious mental health problems often do not receive help in order to support their children [1, 8, 9]. Children of seriously ill parents are at risk of developing their own psychosocial and health problems [10–12]. Hence, children and families at risk should be identified so their needs can be recognized and support can be ensured [13, 14]. There seems to be a knowledge gap regarding factors that may facilitate ill parents seeking help and advice for their parental role in a strained situation. There are social and psychological differences and differences in the needs of families with a somatic ill parent compared to families with a mentally ill parent or a parent with substance abuse, not the least due to different types of shame and stigma related to this variety of family situations. Still, children in these families face some similar burdens and challenges [15, 16], since they all live with a parent that in the vast majority of cases will struggle to meet their children’ needs – at least periodically [2, 17]. Because of this, “children as next of kin” are often dealt with as one group, i.a. concerning legal rights [18] and support.

In Norway, almost all inhabitants are listed with a general practitioner (GP). This doctor is usually the first step into the health care system for everyone. She or he follows the patient during their illness and is the gateway to other areas of the health care service. Hence, a GP is in a good position to identify ill parents in need of support in taking care of their children. Several studies have explored what needs these children may have [19–22], but studies concerning the GP’s facilitating role for the family are few. The GP may lessen the burdens for the families, including the children, in long-term strained situations. The point of departure for the GP who is engaged in the children’s situation is to address the children’s special needs with the parents, and eventually get the parents’ consent to initiate specific follow-up. However, studies have shown that often there are barriers for health personnel to implement this, both in general practice and in hospitals [14, 23, 24]. GPs who considered these children as their responsibility still reported that they either forgot to address the children’s needs, or they were afraid of hurting their vulnerable patients, and possibly increase the parent’s feeling of guilt and shame [14]. Thus, it may be challenging for GPs to address the children’s situation in encounters with their parents. The GP has knowledge about the children’s risk and special needs, but, in the encounters with their parents, they also need to have insight into the parents’ perspective, according to the patient-centered clinical consultation model (McWhinney et al’s [25]). This is the prevailing consultation model taught at medical schools in Norway. It claims that to decide on how to meet the patient’s problem in a useful way for the patient and the family, the GP has to integrate his bio-psycho-social knowledge about the problem with the patients’ perspective, i.e. the patients’ worries and expectations for the consultation. In the encounter, the GP finds a joint agreement together with the patient on how to deal with the issue of concern. In this consultation model, the physician strive to interpret the patient’s illness and problems within his/her own frame of reference, and the patient plays an active part in the consultation [26]. In several studies, the patient-centred approach has been shown to enhance the communication between patient and doctor [27]. Therefore, in the present study, we explored the meaning of the illness for the parents within the realms of the impact of the illness on their own and their children’s everyday life, and their thoughts, feelings and expectations for the GP concerning their children. The aim of this study was to identify important factors for the GP to bear in mind during encounters with ill and substance-abusing parents, to enable the GP to provide appropriate support for the children.

## Methods

The study design is a qualitative analysis of individual semi-structured interviews. We chose a qualitative approach because there were few hypotheses to trace, and we wanted to explore the participants’ thoughts, feelings, expectations and experiences [28]. Individual interviews are appropriate in a situation like ours, when the subject investigated is sensitive [29].

### Data collection

GPs participating in a previous study [14] and GPs in our professional network were asked to recruit patients to the study. The GPs received invitation letters for patients with information and reply forms. They were asked to give these to relevant patients in their practices with the following inclusion criteria: (1) a patient suffering from a mental illness, substance abuse or severe somatic disease; (2) being a parent to one or more children younger than 18 years; and (3) having an illness of sufficient severity to interfere with parenting. If they wanted to participate, the patients returned the reply form with signed consent to the research team. As we do not know how many letters were handed out, we do not know how many that refused to participate. We included participants for a purposive sample with variation in parental problems, gender and rural vs urban residencies. The first author, a female GP, performed the interviews, which were conducted in an office or in the participant’s home according to the participant’s choice. Each interview lasted 45–70 min. The interviews were conducted on the basis of an interview guide developed by the research team. This was used as a support to make sure our core topics were discussed in all interviews. In accordance with McWhinney and Freeman’s [25] perspective, the five core topics were: 1) how the illness might affect their daily life, 2) how it might influence their children, 3) what kind of help was needed for the children, 4) their experience with their own GP, and 5) how the GP might support them in parenting. All informants were interviewed only once, and the interviews were audiotaped, de-identified and transcribed verbatim by the first author. The transcripts were not returned to the participants for comments. We did preliminary analysis during the data collection, and after 12 interviews, we experienced few new relevant themes coming up, and concluded that we had material with sufficient information power for the purpose of the study [30]. From this empirical data, we could achieve a reliable analysis.

### Data analysis

**Fig. 1:**
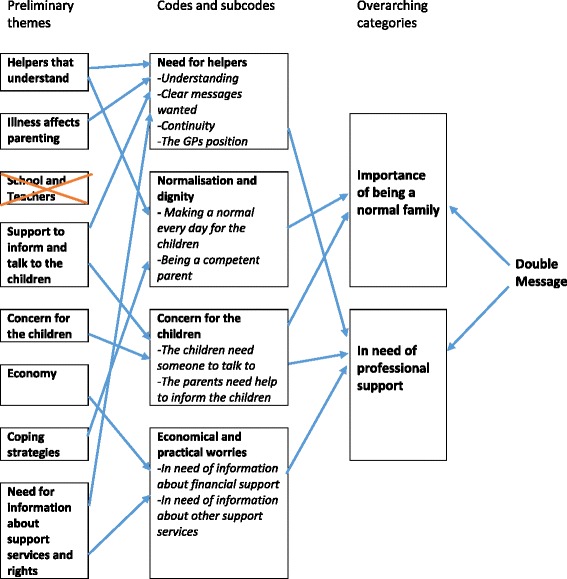
Analysis. The analytic process from preliminary themes over codes/sub-codes to final categories

Data were managed using NVivo 9 software (QSR International, Melbourne, VIC, Australia). We performed a cross-case analysis and used systematic text condensation [31] as an analytical tool (Fig. 1). Starting the analysis, we read the material to get an overview. During this reading, we identified some preliminary themes that were relevant for the aim of the study. In step two, we identified meaning units throughout the material and sorted them into four code groups negotiated from the preliminary themes. In step three, we explored the content of these codes and found them comprising different nuances; thus, we split each code group into sub-codes. We made condensates of the content from all sub-codes, and these condensates formed the basis of the results. Finally, the essence of the codes was merged into two overarching categories: the parents’ need of being seen as competent parents and their need of competent helpers. During this last step, we found that the concept of a ‘double message’ was a central topic for the communication between the patient and the helpers. During the analysis, we continuously went back to the full transcripts to evaluate our codes and sub-codes in the context of the interviews [32]. The analytic work was done by FG and MH, in discussion with the other co-authors and experienced researchers in our network to validate the results and find alternative interpretations [33].

## Results

### Participant characteristics

The sample consisted of three men and nine women (Table 1). Two had addiction problems, four suffered from somatic disease and eight had a mental illness. In total, they cared for 28 children. Two of the participants worked part-time, the others were unemployed. Only half of them lived with the other parent. One participant was a healthy father of an eight-year-old boy where the mother had recently died from cancer. Although he was not himself ill, we included him in the study, as he had relevant experiences.

**Table 1:** Participants’ characteristics

Interview	Gender	Parental problem	No of children	Employment	Live with the other parent of their children
1	Female	Addiction problems	3	Part-time	No
2	Female	Addiction problems (+ mental illness)	1	No	Yes
3	Male	Somatic disease	4	No (sick leave)	Yes
4	Male	Mental illness	3	No	No
5	Female	Mental illness	3	No	No
6	Female	Mental illness	2	No	Yes
7	Male	Somatic illness	1	No (sick leave)	No (wife dead of cancer)
8	Female	Mental illness	2	No	Yes
9	Female	Mental illness	2	Part-time	No
10	Female	Mental illness (+somatic illness)	3	No	Yes
11	Female	Somatic illness	3	No	No
12	Female	Mental illness	1	No	Yes

One overarching finding was that the participants presented themselves as coping parents, but at the same time, they expressed a need for parenting support. On the one hand they expressed knowledge about the impact of their illness on family life, and, in spite of this, how they managed to support their children in everyday life. On the other hand, they expressed uncertainty and concern for the children, and that they were in need of help to secure good care for them. This represented a double message: ‘we are coping, but we still need support’. Most of the participants spoke about a long-term, trustful relationship with their GP, and nobody mentioned any adverse experiences. It was important to all participants that the GP knew about their illness, the family situation and their children. It was also useful that the GP was informed about what kind of help they received, both for themselves and for their children, even if the GP was not very involved in the support for the family. In addition, some parents explicitly wanted advices in parenting matters, including how to inform their children of their illness, professional support for the children and information about the support services available.

### The importance of being an ordinary family

The analysis of the material from our talks revealed that it was vital for these parents to be recognized as responsible parents.

#### Making everyday life normal for the children

The participants strived to make an ordinary everyday life for their children, or at least as ordinary as possible. They wanted their illness to take as small a part as possible in their children’s lives. In addition, being able to master family life was a message both to themselves and to those around them that they were coping despite their problems. A substance-abusing mother living with a husband and a son expressed it like this:

*For the last six months, a woman from the child protection has been coming home to me twice a week to take urinary tests. In addition, she does an inspection in our home. I wanted it that way. I want these people to come home to me, to let them see that we manage just as well as our neighbours, despite my problem.*

When they were asked about daily life, most of the participants told similar stories about the routines in their homes; regular meals, afternoon moments doing homework with their children, the children participating in leisure-time activities, etc. In addition, fixed routines seemed to be helpful when the illness caused challenges.

#### Being a competent parent

Parenthood was seen as a sign of normality and social belonging and was, therefore, important for their identity and self-respect. In addition, some were afraid of being judged as incompetent parents with the risk of losing their children. They gave many examples of how they managed well as parents. Some told about specific strategies during relapses or variations in their level of functioning due to illness in order to protect their children. A father of three children in a family where both parents had bipolar disorder said:

*Now I think we are beginning to cope quite well with the situation, really. We have got our own strategies for many situations that are special for us. For example, if my wife gets very depressed, the deal is that she should see a therapist. In addition, she will have time to recover. The agreement is that she must not let it affect the children (that she is depressed). It is better that she withdraw for a while. It is the same with me; if something occurs or if a symptom shows, we have strategies to handle it. It works very well.*

### In need of professional support

To be able to secure the quality of life they wanted for their children, most of the participants said that it was important for them to have professional support from helpers that knew their situation, including the social and family setting. All participants stated that the doctor was an important person, but their involvement could vary. They appreciated that the GP had a good knowledge of the family and the support services, and worked as a referring authority.

#### Counselling and support from a helper close at hand

Many participants had a trusting relationship with one professional helper. This helper often provided a continuity of care and had a strong personal involvement. It was important for the parents to have easy access to these helpers when they needed support. Many of them mentioned the GP as one of these helpers, but for some it was a cancer nurse, a psychologist or a family therapist. The father of an eight-year-old boy, where the mother had died recently of cancer, expressed their experience like this:

*Support from the GP, a cancer nurse or health visitor is really important. To have helpers genuinely interested in helping you and not just doing a job because it is their duty to do so. You tell more to a person you know and trust than to a person you see only once. These helpers have been there through all the illness. It started with the GP; the GP has been there all the time and it is there you go if new troubles come up.*

From a trusted helper, the parents could tolerate more direct speech, and accept alternative viewpoints and corrections. A strong and trustful alliance also made it easier to involve the children’s situation in the talks. Some informants expressed that they wanted individualised and concrete advices concerning their children’s situation. If they had a trusted helper from another profession, the ill parents’ need to talk with the GP about the children was less. However, all informants welcomed the GP to ask about their children. An ill father of four with cancer explained it like this:

*I am very pleased with my GP, but the only thing he has done concerning my children is to help referring them to the Child and Adolescent Psychiatric Ward. I talk about my children’s situation with a cancer nurse in the community. That is enough for me now.*

Many ill parents, especially those with drug addiction or severe mental illness, received support from different professions. For them, close cooperation between the helpers was important. Scheduled multidisciplinary meetings were mentioned as an effective way of sharing information. By participating in these meetings the GP obtained valuable information and could contribute with information based on his or her knowledge about the ill parent and the family. A mother who had a personality disorder and was the solo parent of two children said:

*My GP is very active participating in collaborative meetings. Then she gets more information about my situation – more than if she just sees me at her office. In those meetings, we talk about almost everything. It is of great importance that the GP participates in these meetings. Otherwise, she would have had no insight. I am not that often at the GP’s office.*

#### The children’s specific needs for information and emotional support

The children’s need for information about their parents’ problems was an ambivalent topic for many of the participants. In hindsight, some parents realized that the children should have been better informed. Some felt it was difficult to know when the best time to inform them was, and they were not sure what information was relevant to share. Some thought it might be best for the children not to know so much about illness and problems. From the participants’ perspective, a helper close by seemed to be the best person to discuss what information to share and how to do it. This helper could also give the children information directly about the parents’ situation, but most preferably together with the parents. A father with bipolar disorder and three children expressed it like this:

*Our experience is that the kids have to trust someone very much to be able to talk about the influence of the illness. It is difficult for someone outside the family to get that role. [….] I am trying as best as I can, and if there is something I do not manage to explain, I can ask my GP about it. Then maybe I can give a better answer. My GP use to be very good finding the right pictures for explanation.*

Many participants said that their children had emotional worries. Some became aware of this in hindsight. The children generally seemed to be reluctant to start talking about the illness or problems at home. They had to be prompted. The parents wanted the children to be offered help to talk about their experiences. Often, the parents, if necessary with counselling from a trusted helper, could be the best conversation partner, but sometimes people outside the family were needed. This could be a helper close by, a teacher, the parents’ psychologist or the GP (among other options). A mother with severe chronic back pain, living with a husband and three children explained it this way:

*[….] because all kids get worried when the mother stays in bed all day, and when they peep into the bedroom she is lying there crying with pain. Of course, my kids got worried. They were terrified. They thought that I would die. They did not see the difference whether I laid there not being able to move because of back pain, or if I had cancer. For them there was no difference. I did not manage to sense their worries. I was staying in bed all day trying to gather strength so that I could do half an hour’s homework with them after school. That was all the energy I had.*

#### Information about support services – a task for the GP

Often, the families and the children were in need of special support. The children could benefit from participating in support groups or other initiatives directed towards the children of ill parents. The ill parent often did not have surplus energy to search for information about services by themselves. They wanted their GP to take the initiative and ask questions about what the family needed. Some parents said they received such information too late. Many of these families had financial problems that affected the children in various ways; for example, what leisure activities they could join in with. The participants stressed that information about financial support was important. A mother with bipolar disorder and two children told us:

*It is important that the GPs have knowledge about where they can recommend us to get help when it comes to the children. Once the doctor knows that we have children, there should be an alarm ringing telling them: ‘Okay, now these kids need to be protected’. The doctor should tell the parents: ‘I have some advices for you, and some helpers you can contact, and here are the phone numbers’, a brochure to hand out or other stuff – I think that can be very helpful.*

## Discussion

It was important for the participants to be regarded as competent parents in ordinary families; however, they realised that their illness affected their parenting. They expressed a need for advices about how to inform their children of their illness and how to talk to them about their experiences. In some cases, helpers who could inform the children and talk to them directly were wanted. Parents needed information about the available support services.

### Discussion of the methodology

In the interviews, we addressed the sensitive theme of how parental illness may affect children. In this situation, parents may want to present themselves with a higher degree of mastery than they actually have, concealing the real problems at home. We can assume that we only get a glimpse into their real lives [34]. However, our goal was not to get insight into the participants’ actual situation. We wanted to learn from these parents about how the GP could meet their expectations in order to give tailored help both for the parents and their children. For that purpose, the interviews contained relevant information.

The interviewed parents had different conditions; mental illness, somatic complaints, and some suffered from substance abuse. However, it was a common challenge that their parenting might be compromised and their children would have some difficulties that they didn’t share with their peers [2, 15, 16]. Performing a cross-case analysis, we aimed to explore how to meet these common challenges. There were few informants with substance-abuse problems, and only three of the twelve informants were male. Concerning these groups, our results must be transferred with care. The participants all contributed with information and reflections on the five main topics in the interview guide.

Most of the participants meant that the GP was an important person for them, and no one mentioned any bad experiences with their GP. This might be related to the fact that they were recruited from GPs and the interviewer was a GP. Probably, not all ill parents experience the same importance of the GP as our informants, and other ill parents might have more adverse experiences. However, our aim was to explore how the GP could meet their expectations for a GP, thus the positive experiences that were reported gave relevant information.

### Discussion of the results

Many of the participants spoke about long-term relationships with their GPs and of many good experiences. Some told explicitly about their relationships with GPs who knew their history, their family and their living conditions. These were GPs that offered regard and care, and whom they trusted. This is in accordance with Ridd et al.’s [35] framework for good doctor–patient relationships seen from the patient’s point of view. The authors distinguish between dynamic factors that develop or maintain the relationship and the depth of the relationship. The depth is a product of the dynamic factors of longitudinal care and consultation experiences, and encompasses what the patients consider to be mutual knowledge, trust, loyalty and regard between the patient and the doctor. A recent study [36] found that the depth of the doctor–patient relationship, as Ridd et al. [35] define it, is associated with more topics raised by the patient and more discussion on emotional and psychological issues in consultations. Skirbekk et al. [37], in their studies of patients’ consultations with GPs, interpret trust as the patient’s implicit willingness to accept the physician’s judgement in matters of concern to the patient. They concluded that in order for the patient to bring psychosocial topics into the encounter, the doctor must achieve a rather open mandate of trust. Our participants considered the relationship with their GP to be good and trustful, and from the abovementioned literature, we may assume this facilitated a talk about the children’s situation in the parent’s encounters with the GP. Thus; these participants can teach us some factors of importance for the GP to bear in mind during the encounters with ill and substance-abusing parents when the aim is to help their children. In the following sub-sections, we discuss three issues from what we consider to be of specific interest for the GP during encounters with these parents.

#### The double message

The interviewed parents spoke about an ordinary everyday life together with their children, but many also talked about circumstances due to their condition that affected their children. On one hand, they said they managed ok; on the other hand, they asked for help. In a previous sub-study, we conducted focus group interviews with adolescents who had ill parents [38]. The stories align well with the stories from the informants in the current study. The adolescents stressed that they took part in ordinary activities just like their peers, but they also told of constraints, duties and obligations caused by parental illness. This ambivalence or balancing act seems to characterize these families regardless of the nature of the parental illness [39]. The GP needs to be aware that there may well be a double message and not immediately take the often first spontaneous answer about ordinary everyday life as being entirely factual. A video study of doctor–patient encounters suggests that the doctor too often lacks curiosity in the patient’s life situation and ends the consultation before exploring these aspects [40]. If the GP recognizes the patient with these sometimes contradictory stories of their lives, it can lead to a shared understanding of the situation, which may contribute to a patient–doctor relationship where the children’s situation is a natural topic [35, 41].

#### Ill parents want to talk to a trusted GP about their children

Our participants wanted their GP to bring up the situation concerning illness, parenting and the home situation. Unless prompted, they might not talk about this at all. Adolescents, as next of kin, tell the same story [38, 42]. Thus, it is important that the GP take the first step to bring up the topic when appropriate. In an interview study we performed with GPs, they spoke about the barriers against bringing up this topic [14]. Professionals’ resistance against introducing the theme about how the children are doing is also documented in other studies [24, 43]. From the present study, it seems that GPs’ fear of touching on the sensitive topic of how patients are coping with parenting is overestimated. All the interviewed parents, having a relationship of trust with their GP, were keen for this topic to be introduced by the GP.

#### Support from a GP concerning parental tasks

All children have basic needs that parenting must address. Illness and substance abuse can interact with these, making parents less able to notice and give their children what they need [1–3, 13]. Relevant links between parental major depressive disorders and offspring psychopathologies are suggested to be the level of parenting skills and how the children cope [44]. Parenting skills are thus an important topic in this setting. For marginalized parents, parenthood can be of significant importance because, among other things, it gives a sense of belonging to ordinary social life [45]. This is in accordance with our results.

Good parenting (positive expressed emotions, support from co-parents) is found to correlate with resilience in youth having a depressed parent [46]. For children, it may be valuable if the GP opens the conversation about parenting in their encounters with the parents, gives advices and refers to special services if needed. The GP must maintain a balance between supporting the parents in challenging parental roles, and securing good care for the children. The GP needs to be able to tell if the parenting is not good enough and be prepared to report to child protection if necessary.

## Conclusion

From the information the parents gave, the GP is welcome to bring up parenting and the children’s situation during their encounters. Ill parents have a double message to GPs: they want to be recognized as responsible and well-intended parents wishing the best for their children, and they need support in parenting. The GP should be aware of and take the time not only to catch up the first spontaneous story about an ordinary everyday family life, but also to explore the parents’ concerns about their children and the level of support needed. Then, the trusted GP can be in a good position to give the parents advices about parenting and ensure follow-up of the children if needed, give information about support services and participate in collaboration with others in the health care system concerning the children.

Gaining more information from substance abusive parents and parents with adverse experiences with their GP might be an interesting aim for further research.
